# Safety and Synergy of Capmatinib Plus Stereotactic Radiotherapy in MET Exon 14-Mutated Non-Small Cell Lung Cancer: A Case Report

**DOI:** 10.7759/cureus.99838

**Published:** 2025-12-22

**Authors:** Othmane Bensalah, Meryem Naciri, Mohamed Ali Boudraa, Fatimaezzahra Aouzah, Fadila Kouhen

**Affiliations:** 1 Radiotherapy, Cheikh Khalifa International University Hospital, Mohammed VI University of Sciences and Health (UM6SS), Casablanca, MAR

**Keywords:** capmatinib, met exon 14 skipping mutation, non–small cell lung cancer, oligometastatic, stereotactic radiotherapy

## Abstract

*MET *exon 14 skipping mutations define a rare subset of non-small cell lung cancer (NSCLC). Capmatinib, a selective MET inhibitor, has shown efficacy, but oligometastatic lesions often require local therapy. Data on combining capmatinib with stereotactic body radiotherapy (SBRT) remain limited. A 73-year-old man with well-controlled type 2 diabetes and a history of smoking presented with a right upper lobe mass, mediastinal lymphadenopathy, vertebral metastases, and a small brain lesion. Biopsy confirmed adenocarcinoma (thyroid transcription factor-1 (TTF-1) +, Programmed Death-Ligand 1 (PD-L1) >80%), and next-generation sequencing revealed a *MET* exon 14 skipping mutation. He received capmatinib (800 mg/day) and denosumab, with SBRT to vertebral metastases and later to the primary lung tumor. Capmatinib was held five days before and resumed five days after each SBRT course. Treatment was well tolerated with no significant toxicity. Imaging showed durable regression of primary and metastatic lesions, with complete resolution of the brain lesion and successful control of isolated oligoprogression. At 24 months, the patient remains alive with no evidence of disease progression. The combination of capmatinib and SBRT was safe and effective, achieving local and systemic tumor control. Temporary suspension of capmatinib during SBRT, along with careful monitoring, optimized tolerability, and outcomes. This case supports the feasibility and potential synergy of precision multimodal therapy in MET-driven oligometastatic NSCLC, highlighting the need for further prospective studies.

## Introduction

Non-small cell lung cancer (NSCLC) remains the leading cause of cancer-related mortality worldwide, accounting for nearly 85% of all lung cancer cases [1]. Despite therapeutic progress, advanced disease continues to be associated with a poor prognosis. Nevertheless, the recognition of an oligometastatic state, defined by a limited number of metastatic sites, has identified a biologically distinct subset of patients who may benefit from aggressive multimodal management combining systemic and local ablative therapies, potentially achieving long-term disease control.

The advent of molecular profiling has revolutionized the management of NSCLC by uncovering actionable oncogenic drivers that guide precision therapies. Among these, *MET* exon 14 (METex14) skipping mutations lead to dysregulated MET signaling through impaired receptor degradation, driving tumor proliferation and survival [2].

Capmatinib, a highly selective MET inhibitor, has demonstrated robust and durable systemic efficacy in patients with advanced METex14-mutated NSCLC, establishing its role as a key component of targeted therapy [3]. In parallel, stereotactic body radiotherapy (SBRT) has emerged as an effective local treatment for primary and metastatic lesions in oligometastatic NSCLC, providing excellent local control with a favorable toxicity profile [4].

The combination of targeted therapies and SBRT represents a promising therapeutic avenue, potentially leveraging synergistic effects between systemic molecular inhibition and local tumor ablation. However, very few studies have specifically evaluated the safety and efficacy of combining capmatinib with SBRT in MET-driven NSCLC, leaving an important gap in clinical evidence.

Here, we report the case of a patient with oligometastatic NSCLC harboring a METex14 skipping mutation, who achieved a dramatic and durable response following a combined approach using capmatinib and SBRT targeting both the primary tumor and metastatic sites.

## Case presentation

A 73-year-old man with a 10-year history of well-controlled type 2 diabetes mellitus and a 25 pack-year smoking history presented with a productive cough and hemoptysis, associated with a 3-kg weight loss and general health deterioration.

**Figure 1:**
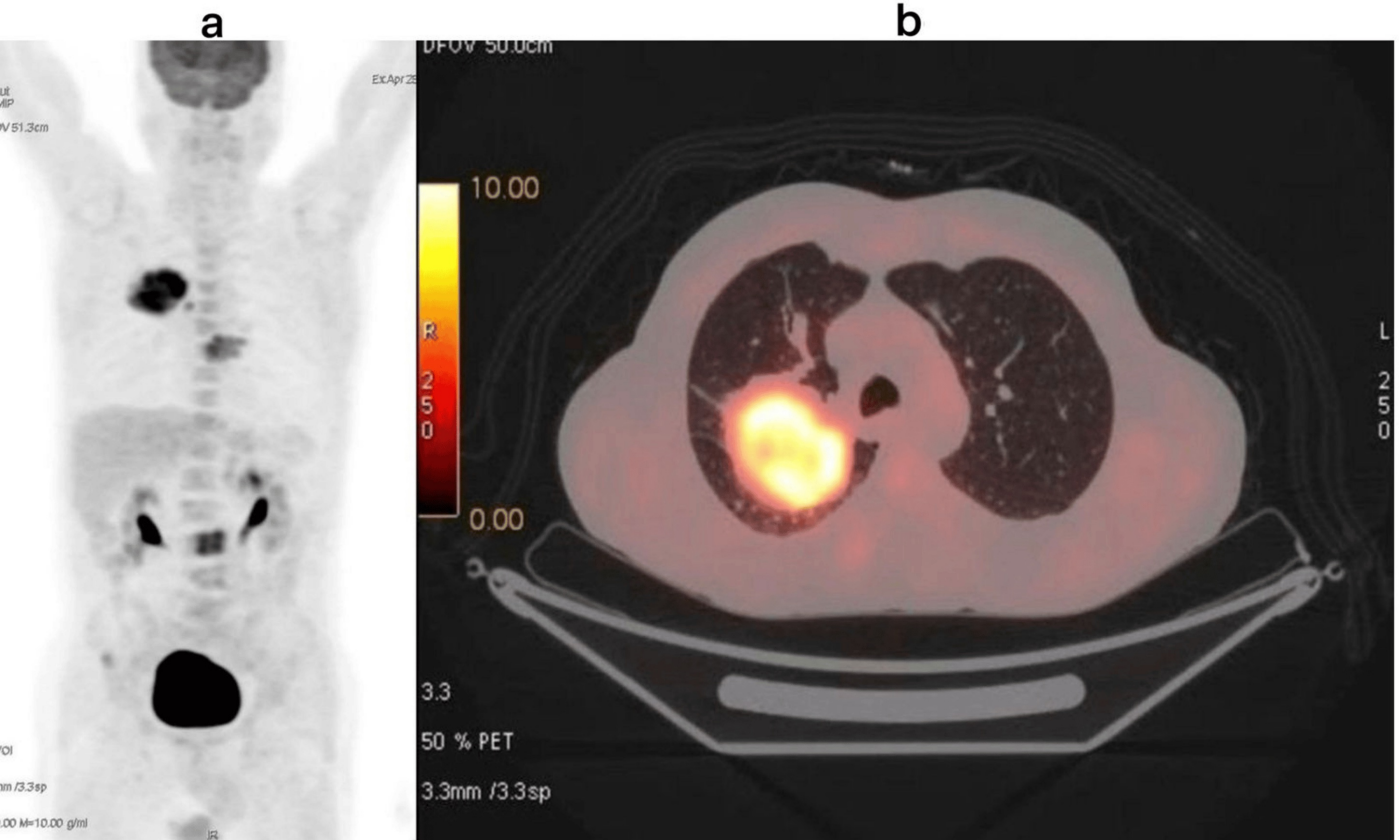
Images from the initial FDG-PET/CT showing the primary lung lesion (a: coronal view; b: axial view) FDG: fluorine-18 fluorodeoxyglucose

Chest computed tomography (CT) revealed a 9 × 7.5 × 5 cm right upper lobe mass, two intertracheobronchial lymph nodes measuring 12 mm, and a lytic lesion of the D7 vertebral body. Bronchoscopy was non-diagnostic, while CT-guided transthoracic biopsy confirmed a moderately differentiated adenocarcinoma, positive for thyroid transcription factor-1 (TTF-1). At presentation, the patient had an Eastern Cooperative Oncology Group (ECOG) performance status of 1 and reported persistent left thoracic pain. Staging with brain MRI demonstrated a small (3 mm) parasagittal enhancing focus. Whole-body fluorine-18 fluorodeoxyglucose positron emission tomography (FDG-PET)/computed tomography (CT) revealed a hypermetabolic right upper lobe lesion (SUV_max_ 13.9), mediastinal lymph node involvement, and bone metastases at T8 and L3 (Figure 1).

Spinal MRI confirmed vertebral lesions at D8 and L4, extending to the posterior elements without spinal cord compression, and additional suspicious lesions at C3 and T4.

The diagnosis of oligometastatic lung adenocarcinoma with limited brain and bone involvement was established. Immunohistochemistry showed CK7+, CK20+, TTF-1+, P40-, and programmed death-ligand 1 (PD-L1) >80%. Next-generation sequencing (NGS) confirmed positivity for a METex14 skipping mutation, with no *EGFR*, *RAS*, or *MEK* alterations.

Based on these findings, a multidisciplinary tumor board recommended targeted therapy with capmatinib (800 mg/day) in combination with denosumab (120 mg every three weeks). The patient initially received SBRT to vertebral metastases (30 Gy in three fractions of 10 Gy), resulting in prompt pain relief. As the brain metastasis was asymptomatic, it was not irradiated.

Before initiating capmatinib, the patient underwent a complete baseline biologic and cardiac assessment, including complete blood count (CBC), renal and hepatic function tests (creatinine, electrolytes, aspartate transferase (AST), alanine transaminase (ALT), bilirubin), and fasting blood glucose to ensure safe management of his diabetes (Table 1).

**Table 1:** Laboratory results with reference ranges SGPT: serum glutamic pyruvic transaminase; SGOT: serum glutamic-oxaloacetic transaminase

	Parameters	Patient Results	Units	Reference Range
Hematology (CBC)	Hemoglobin	14.1	g/dL	13 – 17 (M)
	Hematocrit	42	%	36 – 46
	Red Blood Cells	4.7	T/L	4.0 – 5.2
	Mean Corpuscular Volume	89	fL	80 – 100
	Mean Corpuscular Hemoglobin	30	pg	27 – 32
	Mean Corpuscular Hemoglobin Concentration	34	g/dL	32 – 36
	Total White Blood Cells	7.0	G/L	4 – 10
	Neutrophils	4.1	G/L	1.5 – 7.5
	Lymphocytes	2.3	G/L	1.0 – 4.0
	Monocytes	0.4	G/L	0.1 – 1.0
	Eosinophils	0.2	G/L	0 – 0.5
	Basophils	0.1	G/L	0 – 0.2
	Platelets	247	G/L	150 – 400
	C-Reactive Protein	13	mg/L	< 5
Liver and renal function	Aspartate Transferase (SGOT)	25	U/L	< 35
	Alanine Aminotransferase (SGPT)	30	U/L	< 45
	Total Bilirubin	10	µmol/L	< 17
	Conjugated Bilirubin	3	µmol/L	< 5
	Urea	0.36	g/L	0.15 – 0.45
	Creatinine	8.5	mg/L	6 – 12
Electrolytes	Sodium	140	mmol/L	135 – 145
	Potassium	4.1	mmol/L	3.5 – 5.0
	Chloride	103	mmol/L	98 – 107
	Bicarbonate	25	mmol/L	22 – 28
	Calcium (total)	2.35	mmol/L	2.15 – 2.50
	Magnesium	0.85	mmol/L	0.75 – 0.95
Metabolites	Total Protein	53	g/L	65 – 80
	Albumin	37	g/L	35 – 50
	Fasting Glucose	1.10	g/L	0.7 – 1.1
	HbA1c (Glycated Hemoglobin)	9.0	%	< 5.7 (normal) / < 7 (controlled diabetes)

A 12-lead ECG was obtained to evaluate baseline cardiac rhythm and QT interval. During treatment, these parameters were monitored regularly, with CBC, renal and hepatic function, and fasting glucose assessed at least monthly, and ECG repeated if clinically indicated, allowing early detection and management of any hematologic, hepatic, renal, metabolic, or cardiac toxicity.

Three months after treatment initiation, FDG-PET/CT demonstrated a partial metabolic and volumetric regression of the primary lung lesion (SUV_max_ 9.4 vs. 13.9; volume 16 cm³ vs. 50 cm³) and resolution of bone hypermetabolism. Capmatinib was continued with good tolerance.

**Figure 2:**
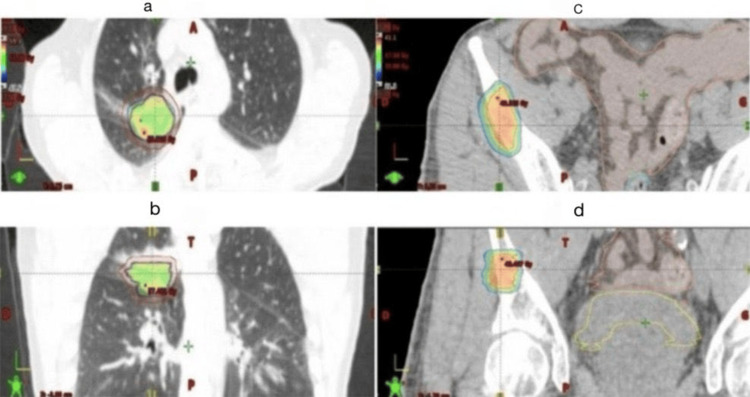
Lung SBRT for the primary lesion (a,b) and SBRT of the pelvic metastatic lesion (c,d) SBRT: stereotactic body radiotherapy

After 10 months, imaging revealed further regression of the primary tumor and complete resolution of mediastinal uptake, with decreased bone activity. Following multidisciplinary reassessment, SBRT to the primary lung lesion (50 Gy in five fractions of 10 Gy) was delivered (Figure 2). Capmatinib was held five days before and resumed five days after SBRT to minimize potential radiosensitization effects.

Subsequent brain MRI demonstrated complete resolution of the previously enhancing lesion. Follow-up FDG-PET/CT confirmed ongoing metabolic regression of pulmonary and bone lesions, with only mild uptake in a right subpleural nodule interpreted as post-therapeutic. A repeat biopsy revealed macrophagic alveolitis without malignant cells. 

Eighteen months after diagnosis, the patient developed isolated oligoprogression in the right iliac crest, which was successfully treated with pelvic SBRT (30 Gy in three fractions of 10 Gy) (Figure 2).

**Figure 3:**
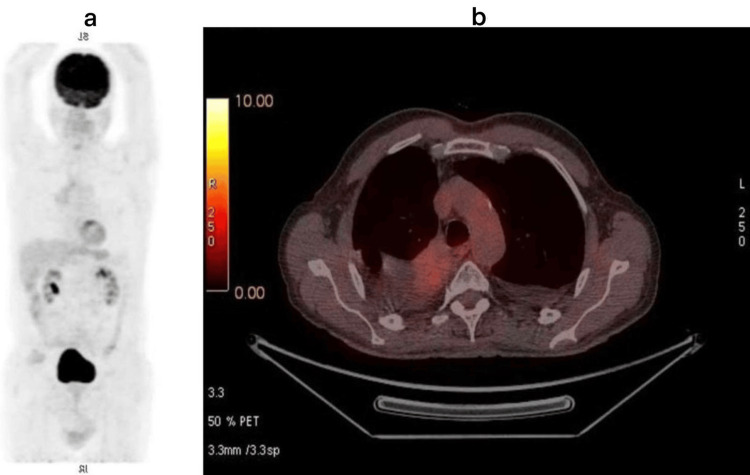
Images from the last FDG-PET/CT showing a complete response (a: coronal view; b: axial view) FDG: fluorine-18 fluorodeoxyglucose

At the most recent follow-up (24 months after diagnosis), the patient remains alive, asymptomatic, and on continuous capmatinib therapy, with no evidence of disease progression, no newly developed hypermetabolic foci on the last FDG-PET/CT imaging (Figure 3), preserved organ function, and excellent tolerance and performance status.

## Discussion

METex14 skipping mutations and other activating alterations in the MET kinase domain represent a relatively uncommon molecular subset of NSCLC, accounting for approximately 3-4% of cases [5]. These alterations are usually mutually exclusive with canonical oncogenic drivers such as *EGFR, KRAS, ALK, *or* RET*, defining a distinct biological and clinical entity with unique therapeutic implications [6]. In the present case, the absence of concomitant genomic aberrations simplified the molecular landscape but simultaneously limited alternative targeted therapeutic options, making MET inhibition the principal systemic strategy [7]. Capmatinib, a highly selective MET tyrosine kinase inhibitor (TKI), has demonstrated robust efficacy in both treatment-naïve and previously treated NSCLC patients harboring METex14 skipping mutations, with overall response rates ranging from 41% to 68% and durable responses in selected individuals [8]. However, despite these encouraging outcomes, systemic therapy alone may not be sufficient to achieve complete and durable control of oligometastatic disease, due to heterogeneous tumor microenvironments, variable drug penetration, and the emergence of resistant subclones [9].

The integration of local ablative radiotherapy, particularly SBRT, with targeted therapy has been proposed as a rational strategy to consolidate systemic responses and improve long-term outcomes [10]. Preclinical evidence indicates that MET signaling contributes to radioresistance through enhanced DNA repair, activation of prosurvival pathways, and promotion of tumor proliferation [11]. Consequently, MET inhibition may sensitize tumor cells to radiation-induced DNA damage, impair repair mechanisms, and enhance apoptosis [12]. Radiation, in turn, can modulate the tumor microenvironment, promote antigen presentation, and potentially synergize with targeted agents to induce systemic antitumor effects [13]. This bidirectional interaction provides a compelling biological rationale for combining MET inhibition with SBRT in MET-driven NSCLC, particularly in the oligometastatic setting where local tumor control can have an outsized impact on survival and quality of life [14].

Clinical evidence for the combination of capmatinib and SBRT remains scarce, limited to isolated case reports and small retrospective series [15]. These reports suggest that sequential or concurrent administration of SBRT with MET inhibition is feasible, can be well tolerated, and may enhance the durability of response [16]. In the present case, SBRT was initially applied to vertebral metastases and later to the primary lung lesion, with capmatinib held five days before and resumed five days after each course to minimize potential additive toxicity while maintaining radiosensitization effects [17]. This approach allowed for careful hematologic, hepatic, renal, and cardiac monitoring, as well as assessment of glycemic control in our diabetic patient, thereby ensuring safety during treatment. The patient experienced no significant hematologic, hepatic, or pulmonary adverse events, supporting the feasibility of this strategy even in individuals with comorbidities.

The clinical outcome in this patient was remarkable, with rapid and durable regression of both irradiated and non-irradiated lesions, sustained disease control of metastatic sites, and normalization of metabolic activity on follow-up imaging. The observation of tumor regression at non-irradiated sites raises the possibility of systemic immune modulation or abscopal-like effects, suggesting that the combination of targeted therapy and SBRT may confer synergistic antitumor activity beyond the directly irradiated volume [17]. Furthermore, the sequential application of SBRT to metastatic and primary sites in conjunction with continuous capmatinib allowed for durable local control, delayed oligoprogression, and minimal toxicity, highlighting the potential of this dual-modality approach in selected oligometastatic patients [18].

This case underscores several important clinical and scientific points. Comprehensive molecular profiling should be systematically implemented in NSCLC, as identification of actionable mutations such as METex14 skipping can dramatically alter the therapeutic trajectory [19]. In the era of precision oncology, combining molecularly targeted agents with highly focused ablative radiotherapy may represent a promising paradigm for managing oligometastatic disease [20]. Careful attention to treatment sequencing, timing of drug interruption, and systematic monitoring of hematologic, hepatic, renal, cardiac, and metabolic parameters is essential to ensure safety and maximize therapeutic benefit. Prospective studies are urgently needed to define the optimal combination schedule, clarify potential synergistic mechanisms, and establish long-term efficacy and safety of SBRT with MET-targeted therapy.

## Conclusions

This case contributes to the growing body of evidence that rational integration of targeted systemic therapy and precision radiotherapy can achieve durable tumor control in MET-driven NSCLC, providing a potential framework for future treatment strategies in this rare but clinically significant molecular subset. By demonstrating the feasibility, safety, and efficacy of combining capmatinib with SBRT, this observation may inform clinical decision-making and stimulate further investigation into multimodal management approaches for oligometastatic NSCLC.
